# Polyethersulfone Blended with Titanium Dioxide Nanoribbons/Multi-Wall Carbon Nanotubes for Strontium Removal from Water

**DOI:** 10.3390/polym14071390

**Published:** 2022-03-29

**Authors:** Tarek Ashraf, Nada Alfryyan, Abdallah M. Ashraf, Sayed A. Ahmed, Mohamed Shaban

**Affiliations:** 1 Chemistry Department, Faculty of Science, Beni-Suef University, Beni-Suef 62514, Egypt; ch.tarekash@gmail.com (T.A.); sanchy_kodo_2010@yahoo.com (A.M.A.); skader_70@yahoo.com (S.A.A.); 2 Nanophotonics and Applications (NPA) Lab, Physics Department, Faculty of Science, Beni-Suef University, Beni-Suef 62514, Egypt; 3 Department of Physics, College of Sciences, Princess Nourah Bint Abdulrahman University, P.O. Box 84428, Riyadh 11671, Saudi Arabia; 4 Department of Physics, Faculty of Science, Islamic University in Madinah, Al-Madinah Al-Munawarah 42351, Saudi Arabia

**Keywords:** polyethersulfone matrix, TNR-MWCNT nanocomposite, nanofiltration membranes, strontium removal, temperature effect

## Abstract

Nanofiltration methods were used and evaluated for strontium removal from wastewater. The phase inversion method was used to create a variety of polyethersulfone (PES)/TiO_2_ nanoribbons (TNRs)–multi-walled carbon nanotubes (MWCNTs) membranes with varied ratios of TNR-MWCNT nanocomposite. The hydrothermal technique was applied to synthesize the nanocomposite (TNRs-MWCNTs), which was then followed by chemical vapor deposition (CVD). The synthesized membranes were characterized by scanning electron microscopy (SEM), transmission electron microscopy, and FTIR. TNR macrovoids are employed as a support for the MWCNT growth catalyst, resulting in a TNR-MWCNT network composite. The hydrophilicity, mechanical properties, porosity, filtration efficiency of the strontium-containing samples, water flux, and fouling tendency were used to assess the performance of the synthesized membranes. The effect of feed water temperature on water flux was investigated as well as its effect on salt rejection. As the temperature increased from 30 to 90 °C, the salt rejection decreased from 96.6 to 82% for the optimized 0.7 PES/TNR-MWCNT membrane, whereas the water flux increased to ≈150 kg/m^2^. h. Double successive filtration was evaluated for its high efficiency of 1000 ppm strontium removal, which reached 82.4%.

## 1. Introduction

Strontium is a rare element that only makes up 0.048% of the earth’s surface [1]. It has oxidation states ranging from 0 to 2, and its most common forms are celestite SrSO_4_ and strontianite SrCO_3_ [2]. The separation of strontium from water may be required in a variety of situations, including drinking water and wastewater treatment. For the first, the Federal–Provincial–Territorial Committee on Drinking Water recommends a maximum allowed content of 7.0 mg/L in drinking water [3], while the US Environmental Protection Agency (EPA) set a health reference threshold of 1.5 mg/L [4]; strontium levels in stream water range from 0.001 to 13.6 mg/L [5]. For wastewater, as produced water accompanied by oil in petroleum production companies contains from a slightly low to a very high concentration of strontium, these strontium traces result from the dissolution of reservoir shale rocks containing mineral clay celestine (SrSO_4_) [6] in produced water accompanied by oil in the form of free water or emulsion. Strontium concentrations in produced water in the Nanyishan oil-field brine of Qaidam, for example, reach 5364 mg L^−1^ [3], which is generally higher than the minimal concentration for industrial exploitation. In addition, a water pollutants study from the Mishrif reservoir (Dubai) revealed a strontium value of 610 ppm [7], and there are two main alternatives for getting rid of wastewater. The first alternative is to filter and treat it to an acceptable environmental range before discharging it into a nearby river or sea, for example, salinity, oil content, dissolved oxygen content (DO), chemical oxygen demand (COD), and naturally occurring radioactive material (such as strontium). The second alternative for wastewater disposal is to inject it into disposal wells in reservoirs that are distinct from drinkable groundwater zones; however, strontium can react with reservoir water and develop scales that can block disposal well perforation in this scenario as well. Many concerns have been raised from injecting produced water in disposal wells, which may penetrate to underground water used for drinking and agriculture and cause adverse effect to health. In addition, the wastewater from nuclear reactor plants contains significant quantities of radionuclides such as ^90^Sr^2+^ and ^137^Cs^+^ [8,9], which must be treated before disposal to reduce the amount to acceptable limits. Several methods for separating Sr^2+^ from water have been investigated, including a series of evaporation and condensation to raise the concentration of strontium in water before extraction [1], Sr^2+^ electrodeposition utilizing 18-crown-6 (DCH18C6) [10], ion-exchange adsorption using hydrous crystalline silicotitanate for Sr^2+^ separation [11], and titanate nanobelt membranes as sorbents for ^90^Sr^2+^ [12]. Nanofiltration was utilized as a separation method for strontium in this study. Nanofiltration lies between ultrafiltration and reverse osmosis filtration in properties as reverse osmosis removes particles with a diameter smaller than 0.0001 micron and ultrafiltration separates particles larger than 0.01 micron diameter compared to nanofiltration membranes, which isolate particles in the range of 0.01 to 0.0001 micron as their pores are in the range of 1 to 3 nm compared to ultrafiltration pores ranging from 10 to 100 nm [13]. In this regard, several attempts to extract Sr^2+^ via nanofiltration have been made, including the use of hydraulic pellet coprecipitation microfiltration (HPC-MF), which has given encouraging results for Sr^2+^ separation [14] and Sr^2+^ removal using chitosan-modified graphene oxide [15]. Better results were obtained by using MWCNTs-interconnected GO hybrid membranes [16]. Ethylenimine oligomer mixture can be used for increasing the strontium rejection by complexing the oligomer with wastewater before using the nanofiltration [17]; also, new adsorbents derived from almond green hull were used for enhancing the separation of strontium [18], To decrease fouling caused by scales formed from divalent cations such as (Mg^2+^, Ca^2+^, Sr^2+^, and Ba^2+^), polyelectrolyte multilayer NF can be employed as a preliminary step before reverse osmosis [19]. The filtration efficiency of TiO_2_-doped ZrO_2_ nanofiltration membranes reached 99.2% [20]; Shaban et al. designed a nanofiltration membrane made of polyethersulfone (PES) and titanium dioxide nanoribbons/multi-walled carbon nanotube nanocomposite, which had high efficiency in water desalination, with a NaCl salt rejection rate of 99% [21]. The efficiency of water filtration from strontium using TiO_2_ nanoribbons/multi-walled CNT nanocomposite blended polyether sulfone membranes with varied ratios of TNR-MWCNT and at various temperatures was investigated in this study for the first time. The morphologies and the structures of the membranes are investigated. The performance of the produced membranes was evaluated using hydrophilicity, filtering efficiency of strontium-containing samples, water flow, and fouling propensity.

## 2. Experimental Details

### 2.1. Materials

Polyethersulfone PES (ultrason E6020P), MW = 58,000 g/mole, was from BASF Company, DMF (N, N-dimethylformamide) was from Sigma Aldrich-Germany, titanium dioxide powder TiO_2_ ferric nitrate Fe(NO_3_)_3_·9H_2_O, cobalt nitrate Co(NO_3_)_2_.6H_2_O, and aluminum nitrate Al(NO_3_)_3_·9H_2_O were from Loba Chemie, India, hydrochloric acid 36.6% was supplied by scharlau from SDFCL, India, and sodium hydroxide NaOH was from ADWIC Egypt.

### 2.2. Synthesis Titanium Nanoribbons (TNR)

Gradually, 10 g of TiO_2_ powder was added to one liter of sodium hydroxide (concentration 10 Molar) with continuous stirring for five hours; then, the solution was heated in a Teflon-lined stainless steel autoclave for 170 °C for 24 h to form sodium titanate nanoribbons. After cooling at 15 °C, the produced powder was washed with dilute HCl (1 Molar); then, it was filtered and rinsed with distilled H_2_O and dried over 60 °C for 7 h for the formation of hydrogen titanium nanoribbons from sodium titanate nanoribbons; finally, it was calcined at 350 °C for five hours for dehydration into TiO_2_-B [22].

### 2.3. Synthesis of Catalyst TNR-MWCNT Nanocomposite Powder

The catalyst for the growth of carbon nanotubes is synthesized by dissolving the following chemicals in 100 mL of water: TNR, ferric nitrate, cobalt nitrate, and aluminum nitrate in the ratios of 10:20:20:50, respectively. Then, ammonia is added while stirring until pH 8 is reached and precipitation forms. After complete precipitation, the precipitated product was filtered and dried at 40 °C for 6 h; then, it was calcined at 450 °C for 6 h [23]. Tubular chemical vapor deposition (CVD) with functionalized TNRs was utilized for the growth of MWCNTs as a catalyst using C_2_H_4_ (carbon source) and N_2_ (carrier gas) with a ratio of 1:10 *v/v* at 700 °C for 50 min. Then, the product prepared was purified by adding the produced powder to 20 mL of nitric acid (8 molar) and sonicated for 5 h, and then, 20 mL of hydrochloric acid (5 molars) was added after sonication and refluxed at 120 °C for 3 h. Finally, the product is filtered and rinsed using distilled H_2_O and dried at 75 °C for 24 h [24].

### 2.4. Fabrication of PES/Nanocomposite Blended Membranes

Membranes were produced by the phase inversion method by adding nanocomposite powder (TNR-MWCNT) to 50 mL of DMF gradually [25]. The quantity of added nanocomposite powder was 0.1, 0.5, and 0.7 g to prepare the 0.1 PES/ TNR-MWCNT membrane, 0.5 PES/ TNR-MWCNT membrane, and 0.7 PES/ TNR-MWCNT membrane while stirring over 3 h; then, it was completed by DMF to 100 mL. After stirring for 6 h to form a homogeneous emulsion, 17.5 gm PES tablets were gradually added to the emulsion over an ultrasonic vibrator at 45 °C and stirred for 12 h. Then, they were left in the refrigerator for 12 h to expel air bubbles before being cast on a clean glass plate of 150 µ thickness using a thin film casting device. Afterwards, they were immersed in a water bath, and the casted membranes were removed and dried in the air [26].

### 2.5. Characterization Techniques

#### 2.5.1. Hydrophilicity of Synthesized Membrane

The contact angles of the membranes before and after adding the nanocomposite were measured using the sessile drop method to determine the nanocomposite addition effect on hydrophilicity.

#### 2.5.2. Morphology of Synthesized Membranes

A field emission scanning electron microscope FE-SEM (Model FEG 250, Quanta, Tokyo, Japan) was used for characterization of the synthesized membranes and to define the effect of adding nanocomposite and its concentration on PES membranes, and the transmission electron microscope (TEM, Model 2010, JEOL, Tokyo, Japan) was used to scan the synthesized MWCNT-TNR nanocomposite.

#### 2.5.3. Fourier Transform Infrared Examination

Pure PES membrane and PES blended with nanocomposite membranes were examined using FTIR (Bruker—Vertex 70, Bruker Optics Inc., Billerica, MA, USA).

#### 2.5.4. Mechanical Properties and Porosity

To assess membrane tensile strength and elongation, a mechanical testing system (INSTRON-5500R, INSTRON, Norwood, MA, USA) was employed. The gauge length and breadth of the dumbbell tensile specimens were 6.2 mm and 0.16 mm, respectively. After a membrane specimen had been inserted between the grips of the testing equipment, the tensile strength and elongation were determined. The measurements are within 5% of each other’s accuracy.

Porosity was determined by immersing the manufactured membranes in distilled water. Then, the surplus water was filtered out using filter paper to determine the weight of the wet membrane (W2). After drying for 24 h at 85 °C, the membrane was weighted to determine W1 (dry membrane) [27]. Membrane porosity is calculated using Equation (1):

(1) $$\mathrm{Porosity}=\frac{W1-W2}{A\times I\times \mathrm{dw}}$$

Here, A is the membrane surface area (m^2^), I is membrane thickness (m), and dw is water density (998 kg.m^−3^).

### 2.6. Membrane Performance

#### 2.6.1. Filtration Efficiency

The water sample contains 100 ppm of strontium chloride, which was prepared by dissolving 0.1 gm of strontium chloride in 1 L of distilled water and stirring for 2 h. Then, filtration was carried out using a pressurized system as in Figure S1 (Supplementary Data). The system contains a peristaltic pump to suck a strontium solution sample and pump it to the membrane cell at a 45 psi discharge pressure. The membrane filter cell has an inlet, outlet, and vent that expels water not passed through the filter to maintain pressure in the required range.

#### 2.6.2. Relationship between Concentration and Absorbance

Different concentrations of strontium were prepared. Then, the absorbance of its solution was measured using a spectrophotometer (Lambda 950 UV/VIS, PerkinElmer, Boston, MA, USA) to determine the relationship between absorbance and concentration.

#### 2.6.3. Evaluation of Filtration Efficiency Using Different Membranes at Different Temperatures

Filtration of a sample (100 ppm Sr^2+^) using a filtration apparatus with three different membranes (0.1, 0.5, 0.7) PES/TNR-MWCNT at temperatures of 30 °C, 45 °C, 60 °C, 75 °C, and 90 °C was performed, and absorbance was measured using a Perkin Elmer spectrophotometer to determine the filtration efficiency using the following equation:

(2) $$\mathrm{Filteration}\;\mathrm{Efficiency}\;=\frac{\mathrm{Co}-C}{\mathrm{Co}}\times 100$$

where C_o_ is blank absorption and C is permeated solution absorption.

In addition, water flux at a discharge pressure of 45 psi was measured by observing permeated quantity every 30 min, with a reduction in permeate quantity for the first 60 min, and then, a constant water flux was attained [28]. Water flux was calculated using the following equation:

(3) $$\mathrm{Jw}=\frac{M}{A.\Delta t}$$

where J_w_ is water flux (kg/m^2^.h), M is the permeate water weight (kg), A is the area of membrane (m^2^), and ΔT is consumed time (h).

#### 2.6.4. Evaluation of Successive Filtration

To check if it can result in more filtration efficiency, double successive filtrations were carried out. The filtration efficiency was evaluated by the spectrophotometric measurements for the single filtration and double-successive filtration in a comparative manner.

#### 2.6.5. Fouling Tendency

The fouling tendencies of membranes were investigated by a dynamic method. To utilize as an excellent fouling agent, a solution of 8000 mg/L milk powder was made. The first filtration using synthesized membranes and pure water was performed for 30 min at 45 psi, and water flux was recorded (J_w1_) Then, a synthesized solution was used to perform filtration at the same parameters of time and pressure using a solution of 8000 mg/L milk. Then, the membranes were washed using deionized water and repeated filtration using pure water while recording related water flux (J_W2_). The flux recovery ratio (F_RR_) is obtained by [29]:

F_RR_ = Jw_2_/Jw_1_ (4)

## 3. Results and Discussion

### 3.1. Hydrophilicity of Synthesized Membrane

The contact angles of the synthesized membranes were measured using the sessile drop method [30], which involved measuring the angle between the water droplet and the dry membrane surface immediately after a water droplet was dropped on the membrane surface, then repeating the process five times and recording the average of the readings. The contact angles for pure PES, 0.1 PES/TNR-MWCNT, 0.5 PES/TNR-MWCNT, and 0.7 PES/TNR-MWCNT membranes were 84, 81, 55, and 47, respectively [31]. This indicates that hydrophilicity increases by an increasing percentage of embedded nanocomposite in the PES matrix.

### 3.2. Morphological Properties

#### 3.2.1. TNRs/MWCNTs Nanocomposite

**Figure 1:**
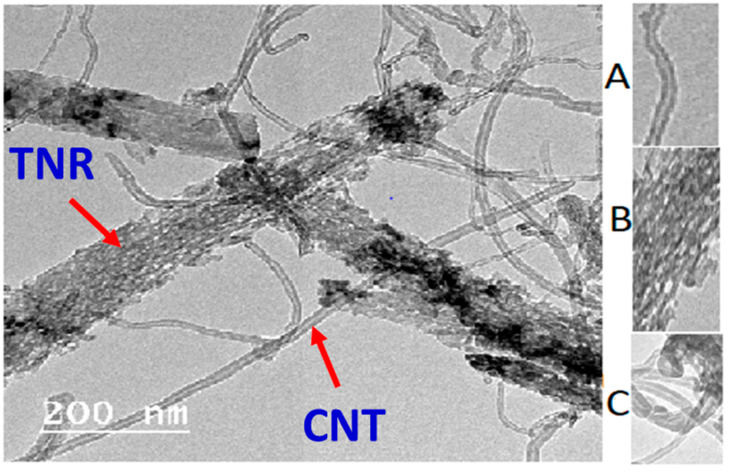
TEM image of nanocomposite with magnified parts for (**A**) MWCNTs, (**B**) nanopits distributed on nanoribbons surface, and (**C**) networking between nanoribbons and nanotubes.

Transmission electron microscopy (TEM) of a nanocomposite reveals wide, long, and straight titanium nanoribbons with nanopits dispersed across the surface [32] that serve as a substrate for the growth of multi-walled carbon nanotubes (MWCNTs). MWCNTs having inner and outer tube widths of 7 to 10 nm and 18 to 20 nm, respectively, and average nanoribbon widths of 15 to 150 nm are shown in Figure 1A. Figure 1B illustrates the distribution of nanopits on the TiO_2_ surface, while Figure 1C illustrates the networking of MWCNTs and spongy nanoribbons.

#### 3.2.2. Pure PES Membrane

**Figure 2:**
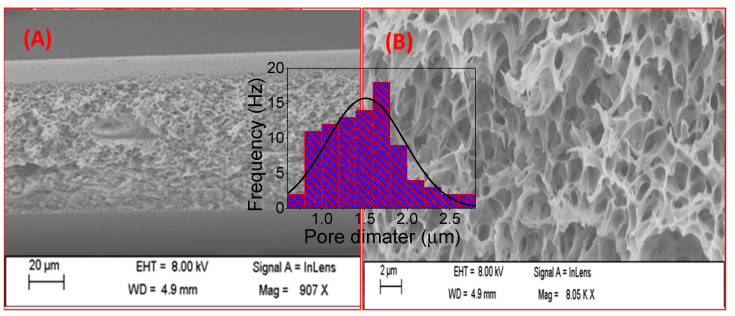
SEM images of pure PES membrane; (**A**) Cross-sectional view and (**B**) top view. The inset histogram showed the pore diameter distribution of the PES.

SEM examination of a pure PES membrane reveals the development of a top layer that is denser and less porous, while the sub-layer has more micro pores, as shown in Figure 2A. The total thickness of the pure PES membrane ranges from 110 to 120 µm. The top view, as shown in Figure 2B, depicts the distribution of micro pits on the surface. The inset histogram showed the pore diameter distribution, whereas the average pore diameter is 1.51 μm. The difference in density and porosity between the top and sub-layers was caused by the solvent/non-solvent exchange mechanism, in which after the casted membrane was soaked in a distilled water bath, the water-immiscible polymer (PES) began to create repulsion forces with water particles, causing the coagulation and precipitation of membrane polymer particles. Simultaneously, the immiscibility of DMF in water caused the partial diffusion of DMF particles out of the casted membrane’s polymer matrix, which was replaced by water particles, causing repulsion between polymer nuclei to the outer boundaries, resulting in a denser, less porous top layer and more porous sub-layer [33,34].

#### 3.2.3. TNRs-MWCNTs/PES Membranes

**Figure 3:**
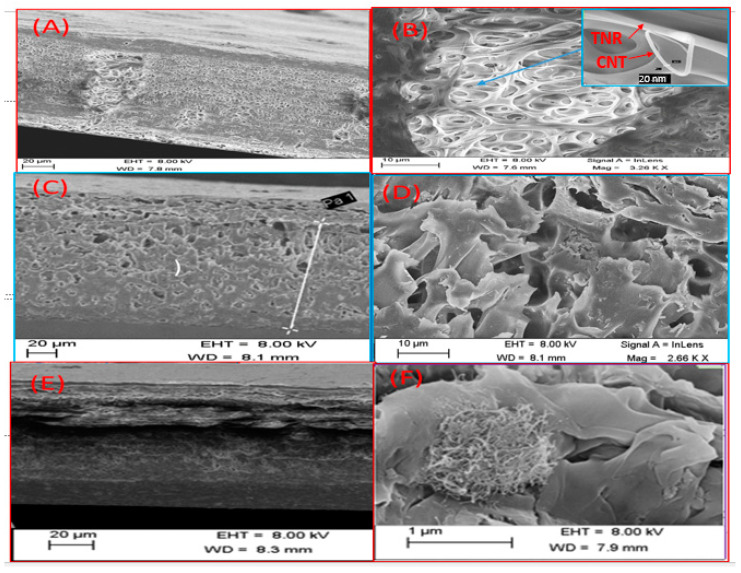
SEM images for (**A**,**B**) 0.1 PES/TNR-MWCNT, (**C**,**D**) 0.5 PES/TNR-MWCNT, and (**E**,**F**) 0.7 PES/TNR-MWCNT membranes.

Figure 3A depicts a cross-sectional view of the 0.1 PES/TNR-MWCNT membrane, showing that the top layer is denser and less porous than the PES membrane, while sub-layer macrovoids occur with an asymmetric distribution along the membrane thickness. The formation of macrovoids can be attributed to the hydrophilic nature of the added nanocomposite, which supports mass transfer rates of solvent and non-solvent [34], allowing water particles to pass through the hydrophobic polymer matrix faster, resulting in repulsion forces that cause macrovoids to form. Figure 3B shows MWCNTs grown on the TNRs macrovoids for the 0.1 PES/TNR-MWCNT membranes. The inset image showed the growth of CNT with a diameter of 20 nm on the surface of TNR. In cross-sectional SEM images for 0.5 PES/TNR-MWCNT, the nanocomposite distribution increases, resulting in the formation of more macrovoids that extend along with the membrane thickness, as shown in Figure 3C,D. This can be attributed to an increase in the hydrophilic nanocomposite, which increases solvent/non-solvent diffusion. In the case of 0.7 PES/TNR-MWCNT, Figure 3E shows that the high concentration of nanocomposite, which leads to high viscosity, resulted in the formation of multilayers. In addition, it resulted in the accumulation of nanocomposite at certain points across the PES matrix, as shown in Figure 3F. From Figure 3A,C,E, the average thickness of the PES/MWCNT-TNR membranes is 140 µm. The increase in skin layer thickness to 140 µm is attributed to a decrease in solvent/non-solvent diffusion due to the increased viscosity. A large number of pores of various sizes were produced.

**Figure 4:**
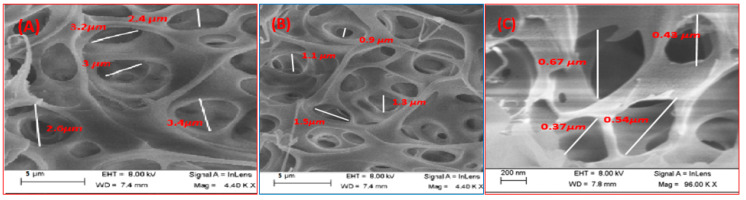
SEM images for (**A**) 0.1 PES/TNR-MWCNT, (**B**) 0.5 PES/TNR-MWCNT, and (**C**) 0.7 PES/TNR-MWCNT membranes with indexed pores diameter for each membrane.

Figure 4 showed top view SEM images for 0.1PES/MWCNT-TNR, 0.5 PES/MWCNT-TNR, and 0.7 PES/MWCNT-TNR membranes. The pore density was less in membranes containing nanocomposite than in pure PES. The pore size of each membrane was measured with a typical sample position. The SEM images in Figure 4 revealed that as the amount of nanocomposite in membranes rose, the average pore size decreased. The sizes of the pores are in the range 2.4–3.4 µm, 0.9–1.5 µm, and 0.37–0.67 µm for the 0.1, 0.5, and 0.7 PES/MWCNT-TNR membranes, respectively. The mean pore areas are 6.9 µm^2^, 0.97 µm^2^, and 0.152 µm^2^ for 0.1, 0.5, and 0.7 PES/MWCNT-TNR membranes, respectively.

#### 3.2.4. FTIR Spectroscopy

**Figure 5:**
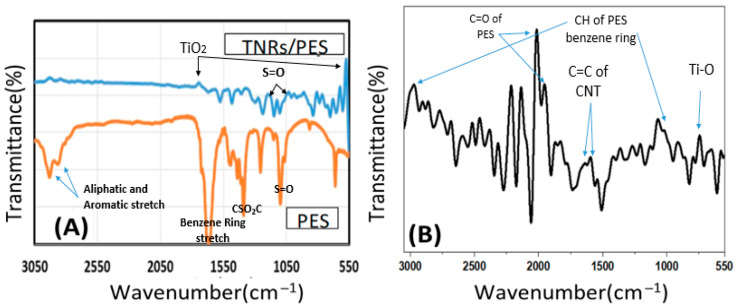
FTIR of (**A**) PES and TNRs/PES, and (**B**) PES/TNRs-MWCNTs membranes.

As indicated in Figure 5A,B, FTIR was used to investigate pure PES, PES/TNR, and PES/TNR-MWCNT membranes. FTIR results reveal the creation of bonds between functional groups of nanocomposite and the PES matrix.

The peaks’ positions and their assignments are listed in Table 1. The wider and lower-intensity beaks of the TNR-PES membrane in Figure 5A were caused by the TiO_2_ suppressing effect [35,36,37,38]. Meanwhile, Figure 5B shows new function groups for PES/TNR-MWCNT, as assigned in Table 1.

The primary FTIR modes of pure PES membrane are S = O at 1150–1307 cm^−1^, CSO_2_C asymmetric stretch at 1322 cm^−1^, and benzene ring stretch at 1587–1489 cm^−1^. Peaks for TiO_2_ asymmetric vibration appear at 600 cm^−1^ and 1700 cm^−1^ for PES-TNR and 560 cm^−1^ for the Ti-O bond. There are peaks at 810 and 3010 cm^−1^ for the C-H of the PES benzene ring, 570 cm^−1^ for the Ti-O bond, and 1750 to 1700 cm^−1^ for C = O stretching for PES/MWCNT-TNR.

#### 3.2.5. Mechanical Properties

**Figure 6:**
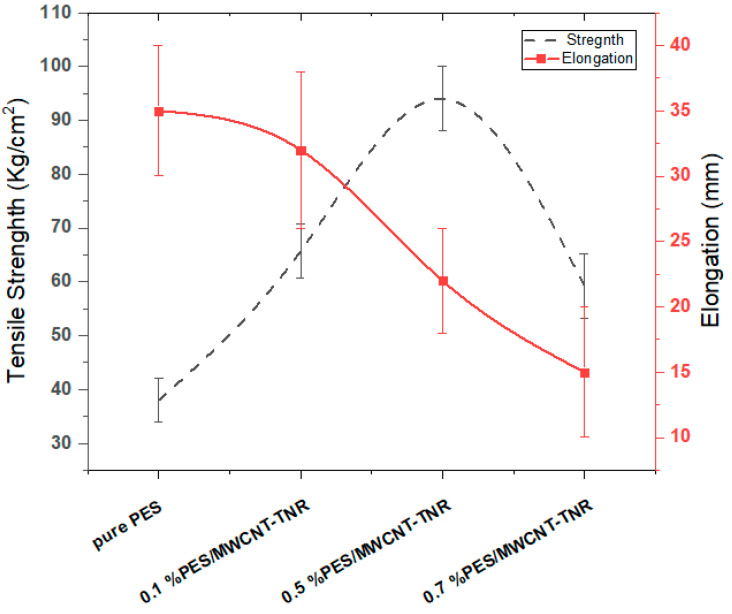
Tensile strength and elongation of the pure and blend membranes.

The membrane’s physical characteristics were improved because of the incorporation of the MWCNT-TNR nanocomposite in the PES membrane. From Figure 6, the greatest tensile strength of the membrane when mixed with 0.5 wt% MWCNT-TNR was 94 kg/cm^2^, which reduced to 59.2 kg/cm^2^ when combined with 0.7 wt% but was still greater than that of pure PES (38 kg/cm^2^). When the membrane was blended with 0.1 wt% MWCNT-TNR, the maximum elongation was 32 mm, as shown in Figure 6. The elongation was reduced to 15 mm at 0.7 wt% MWCNT-TNR because of the high viscosity of the cast solution and the coalescence effect [39]. The blended PES membranes are strengthened by their large surface area, high aspect ratio, and good interaction with MWCNT-TNR [40,41].

The porosity of the prepared membranes was determined using Equation (1). The porosity of PES membrane was 30%. The porosity of membranes decreased as the content of MWCNT-TNR increased. As the composite content increased to 0.1 wt%, 0.5 wt%, and 0.7 wt%, the porosity was reduced to 25%, 13%, and 6%, respectively. A high nanocomposite content cast solution becomes viscous, whereas a low nanocomposite content cast solution becomes less viscous. This delayed membrane development and made the structure denser [41]. As a result, the pore size may be decreased or even blocked. The included nanocomposite also functioned as a nucleation agent during the phase separation process. As a result, membrane nucleation and growth rates increased, making macroporous structure loosening simpler [41].

### 3.3. Evaluation of Membrane Filtration Efficiency

#### 3.3.1. Evaluation of Membranes Filtration Efficiencies

The absorbance spectra of 100 ppm Sr^2+^ aqueous standard solutions were measured using a UV/Vis Perkin Elmer spectrophotometer, which has maximum absorption at 289 nm. This solution will be used as a feed solution for salt rejection evaluation by the membranes. In addition, the absorbance of various strontium concentrations (50, 75, 100, 150, 250, and 500 ppm) was determined, and a plot between absorbance and concentration was drawn. From the plot, a linear relationship appears, which indicates that solution concentrations obey Beer’s Lambert law, so we can apply this method for determining the change in strontium concentration in permeate solutions.

Beer’s Lambert law: A = ϵ L C(5)

where A, ϵ, L, and C refer to the absorbance, molar absorptivity, cuvette length, and solution concentration, respectively.

**Figure 7:**
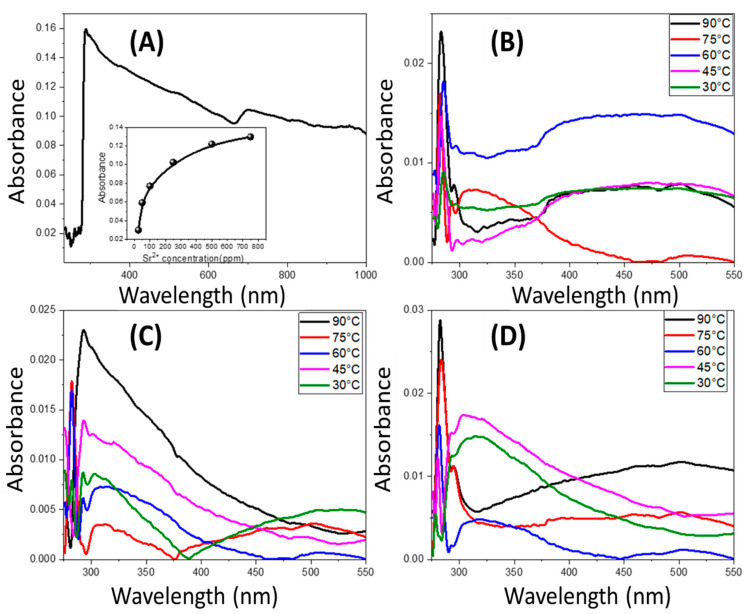
(**A**) Absorbance spectra of 100 ppm Sr^2+^ aqueous standard solution, and (**B**–**D**) absorbance spectra of permeate after filtration using 0.1, 0.5, and 0.7 PES/TNR-MWCNTS membranes, respectively. The inset of (**A**) shows absorbance values versus the strontium concentration at 289 nm.

The absorption spectrum of 100 ppm Sr^2+^ aqueous standard solution and the plot of the absorbance at 289 nm and Sr^2+^ concentration are shown in Figure 7A. The 0.1, 0.5, and 0.7 PES/TNR-MWCNT membranes were used for the filtration of 100 ppm Sr^2+^ aqueous solutions at 30 °C, 45 °C, 60 °C, 75 °C, and 90 °C. The measured absorption spectra using a UV/Vis spectrophotometer in the range of 250 to 550 nm is shown in Figure 7B–D. From absorption curves appear the values of the maximum absorbance for each filtration, and the filtration efficiencies are determined as shown in Table 2.

**Figure 8:**
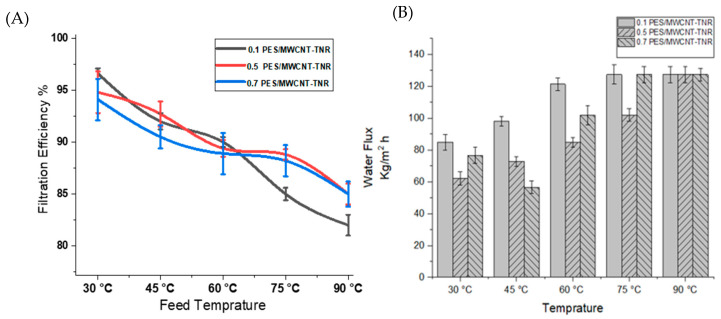
(**A**) Filtration efficiency and (**B**) water flux of the synthesized membranes versus feed sample temperature.

As a result of the analyses, it appears that increasing the filtration temperature of the 0.1, 0.5, and 0.7 PES/TNR-MWCNT membranes decreases the salt rejection ratio. At 30 °C, the 0.7 membrane has the maximum salt rejection compared to the 0.5 and 0.1 membranes, which resulted from the agglomeration of nanocomposite at membrane macrovoids obstructing strontium ions and increasing hydrophilicity. As the temperature rises, the water flux and net salt ion transfer both increase, resulting in the salt rejection drops. It can be shown that temperature enhances the convection, electro-migration, and diffusion of salt ion flux. Temperature causes changes in membrane structural characteristics, solvent viscosity, and solute diffusivity, which all cause these effects [42]. It appears that water flux increases as filtration temperature rises, indicating that membrane pores expand as temperature rises, as thermal expansion coefficients for the PES range of (1.4–2) 10^−4^ k^−1^ [43], while the thermal expansion coefficients (CTE) for CNT and titanium oxide are (1.6–2.6) 10^−5^ K^−1^ [44,45] and (8.4–11.8) 10^−6^ K^−1^ [46], respectively. These differences in thermal expansion coefficient resulted in an increase in PES pores, whereas nanocomposites with lower CTE were unable to compensate, enabling increased water flux as the temperature increases. Figure 8A shows that the salt rejection decreases by increasing filtration temperatures in 0.1, 0.5, and 0.7 PES/TNR-MWCNT. Figure 8B shows that 0.1 PES/TNR-MWCNT has the highest water flux, while the decrease in water flux in 0.5 PES/TNR-MWCNT is attributed to the decrease in pores size of PES by filling nanocomposite. Whereas the slight increase in water flux of 0.7PES/TNR-MWCNT compared to 0.5 PES/TNR-MWCNT may be attributed to the formation of multilayers due to coagulation of nanocomposite [47,48].

#### 3.3.2. Successive Filtration of Highly Concentrated Sr^2+^ Aqueous Solutions

**Figure 9:**
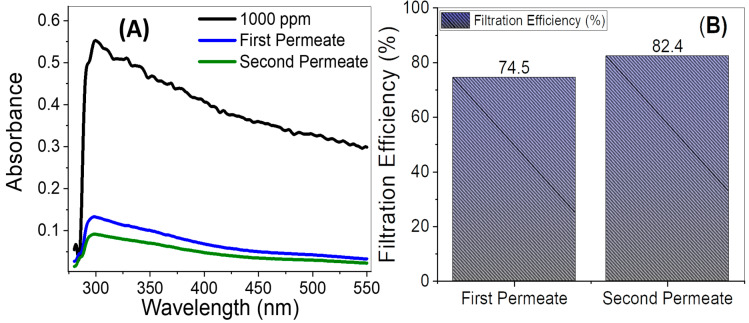
Double filtration-permeate (**A**) absorbance and (**B**) filtration efficiency of 1000 ppm strontium aqueous solution using 0.7 PES/TNR-MWCNT.

The filtration efficiency of a sample of strontium with a concentration of 1000 ppm was evaluated using double successive filtration utilizing a 0.7 PES/TNR-MWCNT membrane and measuring the absorbance of spectra of permeate in the wavelength range of 300 to 1000 nm, as shown in Figure 9A. Figure 9B shows that the filtration efficiency is increased from 74.5% to 82.4% after the double filtration.

#### 3.3.3. Membranes Fouling Tendencies and Impacts

The fouling tendency was investigated by measuring a flux recovery ratio (F_RR_) based on deionized water flux before (J_w1_) and after (J_w2_) filtration of a solution of 5000 ppm of milk powder as a fouling agent solution using synthesized membranes. The higher F_RR_ value indicates a higher antifouling tendency and lower fouling. The F_RR_ values for 0.1PES/TNR-MWCNT, 0.5PES/TNR-MWCNT, and 0.7PES/TNR-MWCNT were 72.7%, 78.7%, and 85.2%, respectively, indicating that the antifouling tendency increased by increasing the ratio of the embedded nanocomposite. The increasing antifouling tendency was attributed to the increased hydrophilicity due to the incorporated nanocomposite [48].

The industrial impact of this research can be concluded as follows. The hydrothermal technique of synthesizing TNR produced nanoribbons with nano pits on their surfaces, which were employed as catalyst support surfaces to grow CNTs on the surfaces of TNRs. There was no agglomeration of nanoparticles while utilizing tubular chemical vapor deposition (CVD) in the synthesis of CNT using C_2_H_4_ as the carbon source and N_2_ as the carrier gas. As a result, the combined approaches utilized in this study construct a TNR-MWCNT network and prevent agglomeration, which is one of the challenges in nanoparticle manufacture. Furthermore, several studies looked at nanofiltration for desalination, but just a few looked into removing strontium from water. Meanwhile, the threat of strontium contamination has increased as a result of high levels of strontium identified in wastewater from the oil and gas industry that exceed permitted limits. Almost Sr-contaminated wastewater is dumped in the sea or injected into wells, posing a risk of contamination of nearby underground water sources. Using our manufactured PES/MWCNT-TNR membrane saves space, increases efficiency, eliminates the need for chemicals, and increases water flow when compared to other standard techniques. Since the produced wastewater has a high temperature after separation from petroleum products in oil treatment stations at petroleum enterprises that utilize demulsifiers and heaters, the influence of feed water temperature on filtering efficacy is significant, as shown in this study. Furthermore, the simple fabrication of PES/MWCNT-TNR membranes will be beneficial in large-scale manufacturing for use in petroleum industries, as well as increasing tensile strength from 38 kg/cm^2^ for pure PES membrane to 94 kg/cm^2^ for 0.5 wt% PES/MWCNT-TNR membranes, which will improve membrane endurance in industrial applications. Meanwhile, this can alleviate the problem of generated water containing excessive levels of strontium, which is a health hazard. This sort of water treatment will be more beneficial at oil-producing off-shore sites where space is limited and the primary means of disposing of generated water is by injecting it into disposal wells, where strontium will develop scales that obstruct well permeability and reduce water injection rate.

## 4. Conclusions

Membranes of PES blended with MWCNT-TNR were synthesized with different ratios of nanocomposite and characterized using TEM, SEM, and FTIR. TEM scanning shows the formation of a network between TNRs and MWCNTs. SEM showed that the top layer of formed membranes is dense and less porous while the sub-layer has macrovoids. SEM also showed the formation of macrovoids in the PES matrix after the incorporation of nanocomposite and the effect of nanocomposite ratio on macrovoid size and distribution, while FTIR scanning proved the formation of a bond between PES and TNR-MWCNT nanocomposite. The pore diameter and porosity are decreased by increasing the ratio of the incorporated nanocomposite. The tensile strength was enhanced from 38 kg/cm^2^ for pure PES membrane to 94 kg/cm^2^ for the 0.5 wt% PES/MWCNT-TNR membranes. The synthesized membranes are used for water filtration of strontium at different temperatures. Salt rejection has decreased as the temperature of the water has risen, while water flux increases by elevating the temperature due to the increasing diameter of PES matrix pores and changes in membrane structural characteristics, solvent viscosity, and solute diffusivity. At 30 °C, the highest filtration efficiency was 96.6% for the 0.7 wt% PES/TNR–MWCNT membrane, compared to 94.8% for the 0.5 wt% PES/TNR–MWCNT membrane and 94.1% for the 0.1 wt% PES/TNR–MWCNT membrane. The highest water flow was 84.4 kg/m^2^.h for the 0.1 wt% PES/TNR–MWCNT, while the lowest was 76.6 kg/m^2^.h for 0.7 wt% PES/TNR–MWCNT. The successive filtration of strontium increased filtration efficiency from 74.5% to 82.4%, which indicated that better efficiency could be achieved by adding multiple filtration steps. By increasing the incorporated ratio of nanocomposite, the antifouling properties of the membranes increased.

## Figures and Tables

**Table 1:** FTIR spectral bands and the related functional groups of PES/TNR-MWCNTS, pure PES, and PES/TNRs membranes.

	Peak Position			Assignment
	PES	MWCNTs	TNR	
PES/MWCNT-TNR membrane	ـــــــــــــ	ـــــــــــــ	570	Ti-O bond
	ـــــــــــــ	ـــــــــــــ	600 and 1700	Asymmetric vibration of TiO_2_
	ـــــــــــــ	960–920	ـــــــــــــ	O-H
	810 and 3010	ـــــــــــــ	ـــــــــــــ	C-H of PES benzene ring
	1170–1110	ـــــــــــــ	ـــــــــــــ	S=O stretching
	ـــــــــــــ	1330–950	ـــــــــــــ	C-O stretching
	1300–1245	ـــــــــــــ	ـــــــــــــ	Stretching peak of C-O-C
	1400 and 1600	ـــــــــــــ	ـــــــــــــ	Aromatic vibration of C-H of PES benzene ring
		1680–1640	ـــــــــــــ	C=C
	1750 to 1700	ـــــــــــــ	ـــــــــــــ	C=O stretching
	3500–2300	ــــــــــــ	ــــــــــــ	-OH of carboxyl group linked to CNT
PES membrane	1150, 1307	ـــــــــــــ	ـــــــــــــ	S=O asymmetric stretch
	1322	ـــــــــــــ	ـــــــــــــ	CSO_2_C asymmetric stretch
	1244, 1260–1000	ـــــــــــــ	ـــــــــــــ	C-O asymmetric stretch
	1587–1489	ـــــــــــــ	ـــــــــــــ	C_6_H_6_ ring stretch
	2886, 2938, 2971	ـــــــــــــ	ـــــــــــــ	Aliphatic and aromatic stretch
TNR/PES membrane	ـــــــــــــ	ـــــــــــــ	560	Ti-O bond
	ـــــــــــــ	ـــــــــــــ	600 and 1700	Asymmetric vibration of TiO_2_
	1170–1110	ـــــــــــــ	ـــــــــــــ	S=O stretching
	1230, 1400, 1600	ـــــــــــــ	ـــــــــــــ	C-O-C Aromatic vibration of C-H of PES benzene ring

**Table 2:** Values of the maximum absorbance and filtration efficiency for 100 ppm Sr^2+^ aqueous solutions at different temperatures using 0.1, 0.5, and 0.7 PES/TNR-MWCNT membranes.

Temperature of Filtration	0.1PES/TNR-MWCNT Membrane		0.5PES/TNR-MWCNT Membrane		0.7ES/TNR-MWCNT Membrane	
	Maximum Absorbance	Salt Rejection	Maximum Absorbance	Salt Rejection	Maximum Absorbance	Salt Rejection
30 °C	0.009012	94.1%	0.00829	94.8%	0.005373	96.6%
45 °C	0.014544	90.5%	0.011714	92.7%	0.012156	92%
60 °C	0.017006	88.9%	0.016885	89.4%	0.015976	90%
75 °C	0.0181522	88.2%	0.017911	88.8%	0.02393	85%
90 °C	0.02508	85%	0.02296	83%	0.02868	82%

## Data Availability

Not applicable.

## Supplementary Materials

The following supporting information can be downloaded at: https://www.mdpi.com/article/10.3390/polym14071390/s1, Figure S1: Peristaltic pump sucking Strontium solution and pump it to membrane cell at 45 psi discharge pressure.
