# Supplementary material for: Polyethersulfone Blended with Titanium Dioxide Nanoribbons/Multi-Wall Carbon Nanotubes for Strontium Removal from Water
Source: Polymers (Basel). 2022 Mar 29;14(7):1390. doi: 10.3390/polym14071390 (PMC9002692; doi:10.3390/polym14071390)
Supplement: Supplementary file 1 [file polymers-14-01390-s001.zip › polymers-1633517-supplementary.pdf]

Polyethersulfone blended with Titanium dioxide nanoribbons/Multi-Wall Carbon Nanotubes for strontium removal from water

Tarek Ashraf[1,2], Nada Alfryyan[3*], Abdallah M. Ashraf[1,2], Sayed A. Ahmed[1], Mohamed Shaban[2,4*]

[1] Chemistry Department, Faculty of Science, Beni-Suef University, Beni-Suef, 62514, Egypt.

[2] Nanophotonics and Applications (NPA) Lab, Physics Department, Faculty of Science, Beni-Suef University, Beni-Suef, 62514, Egypt

[3]Department of Physics, College of Sciences, Princess Nourah bint Abdulrahman University, P.O. Box 84428, Riyadh 11671, Saudi Arabia

[4] Department of Physics, Faculty of Science, Islamic University in Madinah, Al-Madinah Al-Munawarah, 42351, Saudi Arabia.

[*]Corresponding author E-mail: mssfadel@aucegypt.edu (M.S.); naalfryyan@pnu.edu.sa (N.A.)

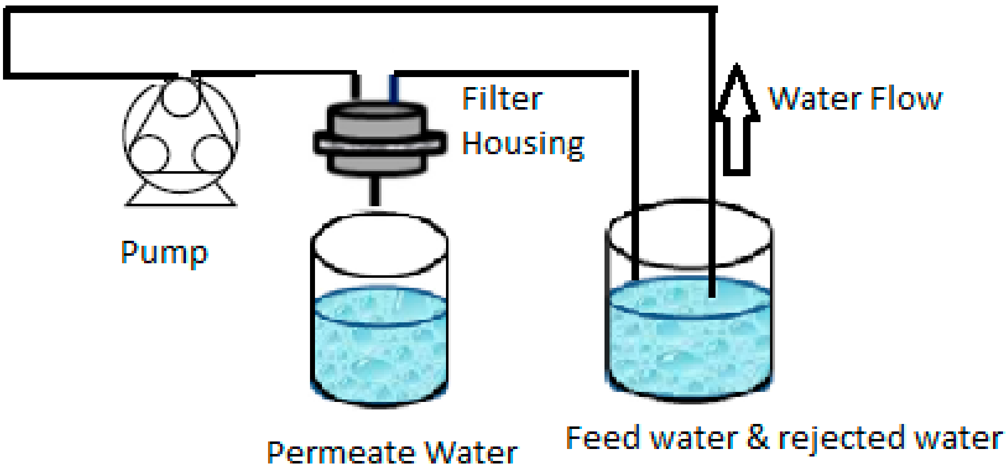

Figure S1. Peristaltic pump sucking Strontium solution and pump it to membrane cell at 45 psi discharge pressure
